# Long COVID-19–related and non-COVID-19 postviral olfactory dysfunction a comparative MRI study focusing on the olfactory cleft and bulbs

**DOI:** 10.3389/fneur.2024.1535699

**Published:** 2025-01-15

**Authors:** Yifan Li, Mengfan Liu, Ruoqi Zhang, Yibei Wang, Jianfeng Liu

**Affiliations:** ^1^Graduate School, Beijing University of Chinese Medicine, Beijing, China; ^2^Department of Otolaryngology-Head and Neck Surgery, China-Japan Friendship Hospital, Beijing, China

**Keywords:** olfactory dysfunction, COVID-19, viral infection, olfactory bulb, olfactory cleft, orbital gyrus distance

## Abstract

**Objective:**

To compare the magnetic resonance imaging (MRI) features of the olfactory cleft (OC) and olfactory bulbs (OBs) in patients with long COVID-19-related (LCOD) and non-COVID-19 postviral olfactory dysfunction (NCPVOD) to explore mechanisms underlying persistent olfactory dysfunction.

**Methods:**

This retrospective analysis included patients diagnosed with LCOD or NCPVOD at the China–Japan Friendship Hospital between February 2023 and July 2024. All patients underwent olfactory psychophysical testing (Sniffin’ Sticks), a visual analogue scale (VAS) for olfactory function, and high-resolution MRI scans of the olfactory pathway. MRI features, including OC opacity, OB morphology, OB volume, and olfactory sulcus depth, were compared between groups. Correlations between MRI findings and olfactory test scores were assessed.

**Results:**

Seventy patients were included (35 LCOD, 35 NCPVOD). LCOD patients had significantly higher OC opacity scores than NCPVOD patients (*p* < 0.001). No significant differences were found in OB morphology, abnormal OB signals, OB volume reduction, or distances between OBs and surrounding structures (*p* > 0.05). LCOD patients had significantly greater right olfactory sulcus depth than NCPVOD patients (*p* = 0.026), with negative correlation to age (r = −0.25, *p* = 0.04). OB volumes positively correlated with TDI and VAS scores.

**Conclusion:**

LCOD patients exhibited greater OC opacity than NCPVOD patients, suggesting OC inflammation may contribute to persistent olfactory dysfunction. Treating inflammation in the OC could improve long-term olfactory outcomes. OB volume reduction was common in both groups.

## 1 Introduction

Since the global outbreak of SARS-CoV-2 in 2019, long COVID-19-related olfactory dysfunction (LCOD) has emerged as a significant clinical challenge. Olfactory dysfunction (OD) is a prevalent and early symptom of COVID-19, with studies reporting that 40–85% (1–3)of COVID-19 patients experience smell loss, often with a sudden onset. This condition has significantly impacted patients’ quality of life (4–7). Studies have shown that the recovery of olfactory function post-COVID-19 is often slow; most patients experience smell recovery approximately 15 (8) to 30 (9) days after infection, but approximately 5.6% (7) to 12.8% (6) of patients exhibit varying degrees of olfactory loss months or years after infection. Indeed, 2 years after being cleared of the infection, up to 2.9% (10) of patients still present with olfactory dysfunction.

Comparatively, postviral olfactory dysfunction unrelated to COVID-19 (non-COVID-19 postviral olfactory dysfunction, NCPVOD) is generally less prevalent and has a more gradual onset. It often results from upper respiratory infections with viruses like influenza or rhinovirus, with partial recovery expected over time. Although both conditions can lead to chronic smell loss, the underlying mechanisms, prevalence, and impacts on the nervous system differ. For example, COVID-19 is thought to affect the central nervous system by targeting sustentacular cells in the olfactory epithelium via ACE2 receptors, leading to indirect neuronal damage and potential inflammation in the olfactory bulb (11, 12). In contrast, NCPVOD primarily affects the olfactory epithelium without extensive neuroinflammatory involvement (13).

While basic differences in prevalence and severity have been established between COVID-19-related and non-COVID postviral olfactory dysfunction (4, 14), there is limited research examining MRI-detectable structural and functional differences in the olfactory system across these two types of dysfunction. The variability in the impact of viruses on the olfactory pathway, affecting both the olfactory epithelium and central olfactory processing regions, adds complexity to understanding these conditions (15). In recent years, MRI has been widely used in the study of the olfactory pathway (12, 16, 17), especially in analysing structural and functional changes in the OC and OB. MRI can clearly depict multiple indicators of such changes, such as the opacity of the OC, the volume and shape of the OB, and abnormal signals in OB (12, 18). To date, however, few studies have compared the MRI findings in patients with LCOD and NCPVOD (19). Therefore, this study aims to compare the MRI findings of the OC and OB between these groups of patients to better understand the differences in their pathological mechanisms and explore potential clinical treatment targets.

## 2 Materials and methods

### 2.1 Study population

This was a retrospective study of patients with olfactory dysfunction who visited the Center for Smell and Taste Disorders in the Department of Otorhinolaryngology, Head and Neck Surgery at China-Japan Friendship Hospital from February 2023 to July 2024. The patients were divided into two groups: the LCOD group and the NCPVOD group. The study protocol was approved by the Institutional Review Board of the China-Japan Friendship Hospital (No.2022-KY-196), and all patients provided written informed consent. The procedures were developed in accordance with the Helsinki Declaration of 1975 and its 1983 revision.

The inclusion criteria for the LCOD group were as follows: prior infection with SARS-CoV-2, COVID-19 positivity was determined based solely on RT-PCR results; postinfection development of symptoms of olfactory dysfunction; disease duration of more than 4 weeks, i.e., the onset of symptoms of olfactory dysfunction persisted for more than 4 weeks (20); and ability to undergo olfactory pathway MRI and other clinical assessments. The exclusion criteria for the LCOD group were a history of long-term olfactory dysfunction; a history of head trauma; severe COVID-19; pregnancy; and lactation.

The inclusion criteria for the NCPVOD group were as follows: upper respiratory tract infection symptoms, negative COVID-19 antigen or antibody tests, or positive tests for other viral infections (e.g., influenza A/B, respiratory syncytial virus, etc.); postinfection development of olfactory dysfunction symptoms; and ability to undergo olfactory pathway MRI and other clinical assessments. The exclusion criteria for the NCPVOD group were identical to those of the LCOD group.

### 2.2 Olfactory psychophysical test

Olfactory function was assessed with the Sniffin’ Sticks Test (21, 22), a psychophysical test conducted with odour-filled felt-tip pens. The pen tip was placed 2 cm below a single or both nostrils.

The test consists of three components: Odour threshold (T): Evaluates the lowest concentration of odour detectable by the patient; Odour Discrimination (D): Assesses the patient’s ability to distinguish among different odours; and Odour Identification (I): Measures the patient’s ability to correctly identify specific odours.

Each component is scored on a scale of 1 to 16, and the total score of the three tests is referred to as the threshold, discrimination, and identification (TDI) score. The TDI score was used to categorize the olfactory function of the patients as follows: normosmia (normal olfaction): TDI score ≥ 30.5; hyposmia (reduced olfaction): TDI score between 16.5 and 30.5; and anosmia (absence of olfaction): TDI score < 16.5.

### 2.3 Olfactory visual analogue scale

In this study, the olfactory function of the patients was also subjectively assessed with a visual analogue scale (VAS). The VAS consists of a 10 cm long horizontal line, with the ends labelled as follows: 0 points, representing complete loss of smell, and 10 points, representing the best olfactory function. Patients were asked to mark a point on the line corresponding to their perception of their olfactory function. A lower score indicates worse olfactory function.

This scale provides a simple and intuitive method to measure a patient’s subjective perception of olfactory function and is commonly used in olfactory research to capture patient-reported outcomes (23).

### 2.4 Olfactory pathway MRI evaluation indicators

#### 2.4.1 Scanning technique

MRI scans were performed within 7 days of the olfactory psychophysical test to minimize variability. All patients underwent MRI examination of the olfactory pathway with a 3 T scanner (Discovery MR750 scanner, GE Medical Systems, United States) equipped with an 8-channel phase-array head coil. A standardized structural MRI protocol was used for all subjects, targeting the left and right OB and covering the anteromedial cranial base with a coronal T2-weighted fast spin–echo sequence with the following parameters: TR/TE = 6660/145 ms; slice thickness = 2.5 mm; matrix size = 320 × 320; 22 slices; flip angle = 142°; average = 3; in-plane resolution = 0.2 × 0.2 mm, and no interslice gap.

#### 2.4.2 MRI evaluation indicators

Measurements were independently performed by two operators, and discrepancies were resolved by consensus to reduce bias.

##### 2.4.2.1 OC opacity score

The OC, located in the upper part of the nasal cavity, was evaluated on coronal T2-weighted images. The OC consists of the lateral wall (the lateral wall of the nasal cavity above the middle turbinate), the superior wall (cribriform plate), and the medial wall (the upper part of the nasal septum). The opacity of the OC was assessed with the Lund–Mackay (24) scoring system: 0: normal, open OC; 1: partially occluded OC; 2: completely occluded OC. Each side was scored separately, and the total score ranged from 0 to 4.

##### 2.4.2.2 OB morphology

According to the protocol by Yan et al. (25), the OB morphology in the posterior tangent through the eyeballs (PPTE) layer was classified into convex types (olivary, circular, and plano-convex) and nonconvex types (banana-shaped, irregular, planar, and scattered). Yan et al. (25) shows differences in olfactory function between olfactory bulbs of different morphologies.

##### 2.4.2.3 Abnormal signals in the OBs

On coronal T2-weighted images, the OB signal intensity was evaluated with reference to the signal from the contralateral straight gyrus. Abnormal signals in the OB included hyperintensities and hypointensities (12, 26). Abnormal signals of the OB include hypo- and hyperenhanced signals. Low signals are often considered to reflect a haemorrhagic focus (26), while abnormal enhancement has been proposed to be due to inflammation of the OBs (27). The extent of the abnormal signal across different OB layers was scored as follows: 0: normal signal, no abnormality in any layers; 1: abnormal signal present in ≤1/2 of all layers; 2: abnormal signal present in >1/2 but not all layers; 3: abnormal signal present in all layers.

##### 2.4.2.4 OB volume

The OB volume was measured with the manual segmentation method (MS). In Annet Viewer software, the contours of the left and right OB were outlined on consecutive coronal T2-weighted imaging slices. The total volume was calculated by summing the areas of all slices and multiplying by the slice thickness (28). According to Hummel et al., the normal OB volume in adults is ≥58 mm^3^ (29), with smaller values indicating OB atrophy. Significant correlations between OB volumes in relation to olfactory function (29).

Due to the lack of established spatial measurements in existing olfactory studies. Therefore, we introduced the new parameters of OB to orbital gyrus and rectus gyrus distances as well as OB to optic nerve distance as part of our exploratory analyses to better understand the anatomical relationships and potential structural changes in the olfactory pathway. Attempts to explore the possibility that spatial variations in the relationship of the OB to surrounding structures reflect differences in the anatomical integrity and functional integrity of the olfactory pathway.

##### 2.4.2.5 OB to orbital gyrus and rectus gyrus distances

**Figure 1:**
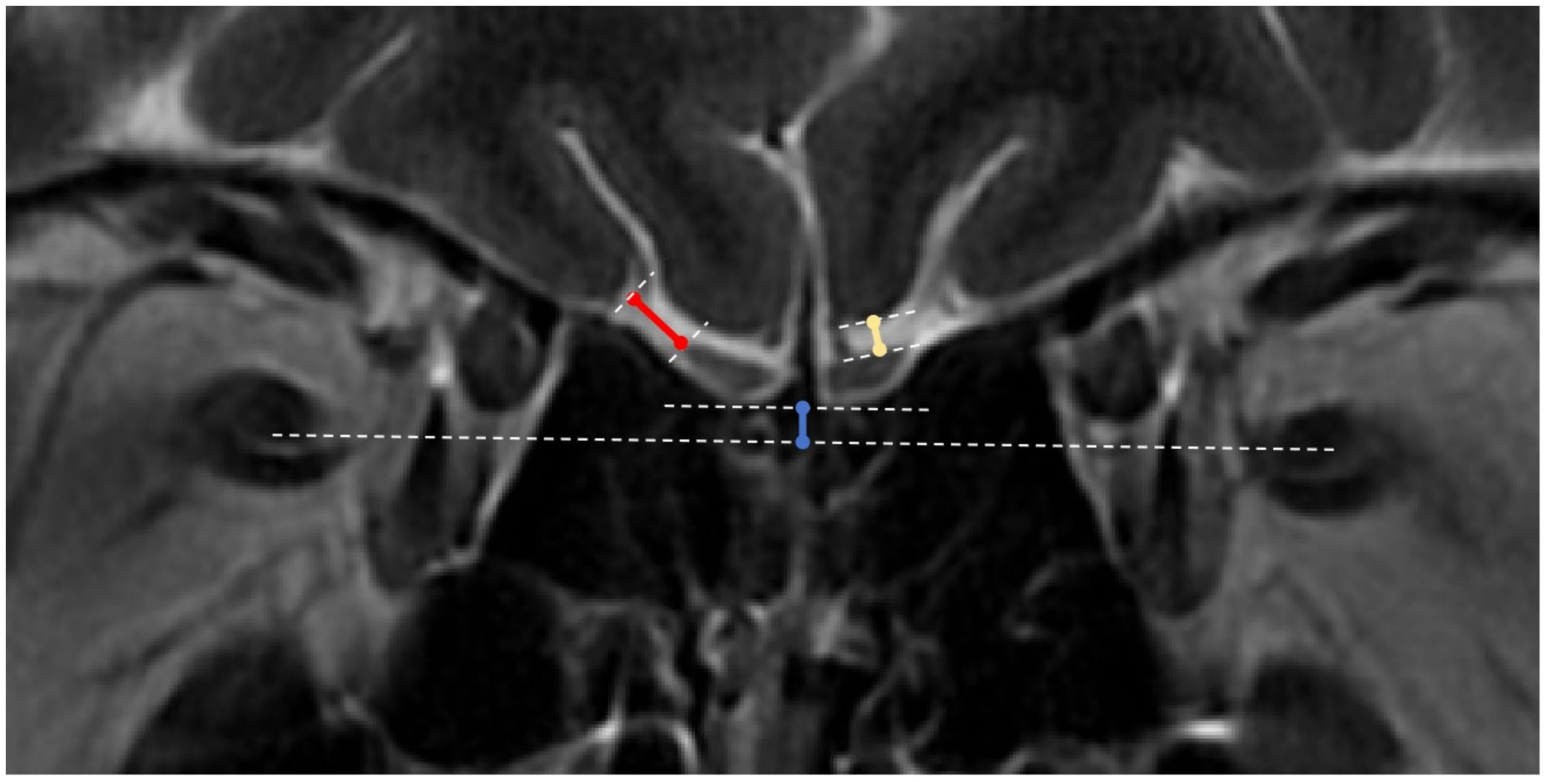
OB to orbital gyrus, rectus gyrus and optic nerve distance measurements. White dotted lines: tangents to the surfaces of the orbital gyrus (OG), rectus gyrus (RG) and olfactory bulbs (OBs), line connecting the bilateral optic nerves, and tangent to the lower ends of the bilateral OBs; blue solid line: distance between the OB line and the optic nerve line; yellow solid line: distance between the OB and the RG; red solid line: distance between the OB and the OG.

These relatively new measurement criteria were obtained from the coronal T2-weighted imaging slice containing the PPTE. Specifically, the shortest distances between the OB and orbital gyrus (OG) and between the OB and rectus gyrus (RG) were measured on this slice (Figure 1).

##### 2.4.2.6 Position of the OB relative to the optic nerve

This is another novel measurement. On the same coronal slice (PPTE), the position of the OB relative to the optic nerve was measured. First, separate lines were drawn connecting the two optic nerves and the two OBs. A perpendicular line was then drawn from the OB line to the optic nerve line, the length of which represents the relative position. If the bilateral OBs were not at the same level, the closer OB was used as the reference (Figure 1).

##### 2.4.2.7 Olfactory sulcus depth

The olfactory sulcus depth was measured on the PPTE slice by drawing a line connecting the surfaces of the orbital gyrus and the rectus gyrus. The shortest distance from the deepest point of the olfactory sulcus to this line was recorded as the olfactory sulcus depth (30). In accordance with Kandemirli et al. (12), a baseline value of 7.5 mm was used, with values <7.5 mm considered shallow. Previous studies (31) have found a positive correlation between olfactory groove depth and olfactory function.

### 2.5 Statistical analysis

For continuous variables, data are presented as the mean ± standard deviation (x ± s) if they followed a normal distribution or as the median (interquartile range) if they did not follow a normal distribution. Independent t-tests was used to compare normally distributed quantitative variables between 2 groups. The Mann–Whitney U test was used when comparing skewed variables between groups. While categorical variables were compared using chi-square tests. Using ANOVA to calculate the side x group interaction effect for the olfactory sulcus depth, OB to RG distance and OB to OG distance. Correlation analyses between olfactory VAS scores and total TDI scores and OC opacity scores, olfactory sulcus depth, OB volume, and other relevant data were performed with Spearman’s correlation analysis. All the statistical analyses were conducted in SPSS version 25.0 (IBM SPSS Statistics) software, with *p* values <0.05 considered to indicate statistical significance.

## 3 Results

### 3.1 Demographic data

A total of 35 LCOD patients and 35 NCPVOD patients were included in the study following application of the inclusion and exclusion criteria. The demographic data of the two groups are shown in Table 1. No significant differences in sex, age, or disease duration were noted between the two groups.

**Table 1:** Demographic data, TDI scores and VAS scores for LCOD and NCPVOD patients.

	LCOD (*n* = 35)	NCPVOD (*n* = 35)	df	Effect size	t/*χ*^2^/Z	*p*
Sex			1	0.086	*χ*^2^ = 0.521	0.631
Female	21 (53.8%)	18 (46.2%)				
Male	14 (45.2%)	17 (54.8%)				
Age	35.86 ± 11.01	39.89 ± 10.67	68	0.185	t = −1.554	0.125
Disease duration (month)	5 (2, 7)	2 (1.5, 6)	68	0.175	Z = -1.465	0.143
Sniffin’ Sticks test						
TDI	21 (14.25, 28)	22.5 (7, 28)	68	0.169	Z = -0.141	0.888
T	4 (1, 5)	2 (0, 4)	68	0.162	Z = -1.355	0.175
D	8 (5, 8)	9 (0, 11)	68	0.021	Z = -0.177	0.859
I	9 (7, 9)	10 (3, 13)	68	0.023	Z = -0.189	0.850
VAS	3 (1.5, 5)	3 (0.75, 5.625)	68	0.035	Z = -0.290	0.637

### 3.2 TDI and VAS scores

There were no significant differences in the total TDI, the T, D, or I or the VAS scores between the two groups (Table 1). In the LCOD group, 26 patients exhibited hyposmia (TDI scores between 16.5 and 30.5), and 9 patients showed anosmia (TDI scores <16.5). In the NCPVOD group, 21 patients exhibited hyposmia, and 14 patients showed anosmia.

### 3.3 OC opacity score

The OC opacity score was significantly higher in LCOD patients than in NCPVOD patients (2 (1, 2) vs. 1 (0, 2), *p* < 0.001; Table 2). Increased opacity was indicative of inflammation. The percentage of patients with OC opacity was 79.4% in the LCOD group and 65.8% in the NCPVOD group, but this difference was not significant (*p* > 0.05).

**Table 2:** Comparison of the OC Opacity Scores of LCOD and NCPVOD.

	LCOD (*n* = 35)	NCPVOD (*n* = 35)	df	Effect size	*χ* ^2^	*p*
Right OC			2	0.347	*χ*^2^ = 8.407	0.015
0	10 (28.6%)	21 (60.0%)				
1	23 (68.6%)	12 (34.3%)				
2	1 (2.9%)	2 (5.7%)				
Left OC			2	0.274	*χ*^2^ = 5.237	0.073
0	7 (20.0%)	14 (40.0%)				
1	27 (77.1%)	18 (51.4%)				
2	1 (2.9%)	3 (8.6%)				
Bilateral OCs			4	0.536	*χ*^2^ = 20.093	<0.001
0	8 (22.9%)	12 (34.2%)				
1	3 (8.6%)	14 (40%)				
2	23 (65.7%)	6 (17.1%)				
3	0 (0%)	1 (2.9%)				
4	1 (2.9%)	2 (5.7%)				

OC, Olfactory cleft.

### 3.4 OB morphology

A total of 17 (48.6%) had convex OBs in the LCOD group versus 23 (65.7%) in the NCPVOD group. The classification of OB morphology in both groups is shown in Table 3, which reveals no significant differences between them. Furthermore, the distributions of OB morphology were not correlated with the total TDI score, VAS score, or disease duration.

**Table 3:** Comparison of OB morphology and internal signals between the LCOD and NCPVOD groups.

	LCOD (*n* = 35)	NCPVOD (*n* = 35)	df	Effect size	*χ* ^2^	*p*
OB morphology						
Convex	17 (48.6%)	23 (65.7%)	1	0.173	*χ*^2^ = 2.100	p* = 0.147
Olive-shaped	4 (11.4%)	12 (34.3%)	5	0.333	*χ*^2^ = 7.776	p** = 0.138
Circular	4 (11.4%)	6 (17.1%)				
Plano-convex	9 (25.7%)	4 (11.4%)				
Nonconvex	18 (51.4%)	12 (34.3%)				
Banana-shaped	11 (31.4%)	9 (25.7%)				
Irregular	0	0				
Plane-shaped	6 (17.1%)	4 (11.4%)				
Scattered	1 (2.9%)	0				
OB signal			3	0.236	*χ*^2^ = 3.889	0.274
Normal	8 (22.9%)	13 (37.1%)				
hypointense	10 (28.6%)	4 (11.4%)				
hyperintense	16 (45.7%)	17 (48.6%)				
Unclear	1 (2.9%)	1 (2.9%)				
OB signal score			3	0.285	*χ*^2^ = 5.679	0.128
0	8 (22.9%)	14 (37.1%)				
1	6 (17.1%)	10 (28.6%)				
2	10 (28.6%)	8 (22.9%)				
3	11 (31.4%)	4 (11.4%)				

OB, Olfactory bulb; p* *p* represents the significance of convex and non-convex between the two groups; p** represents the significance of detailed types between the two groups.

### 3.5 Abnormal signal in the OB

**Figure 2:**
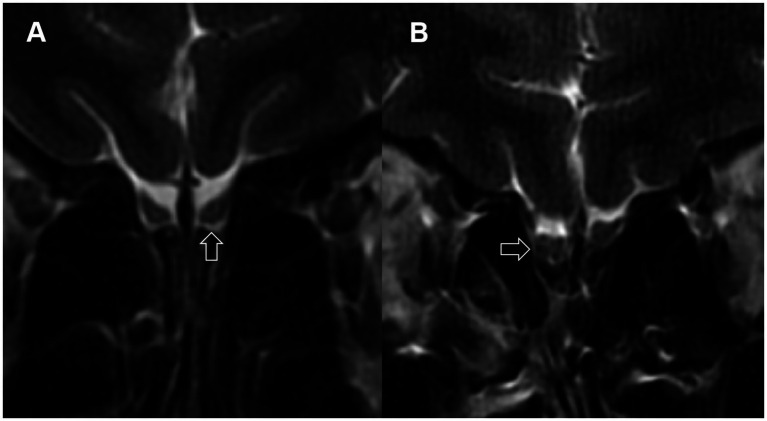
Abnormal signals in the olfactory bulbs. **(A)** Arrow points to a localized high-signal area in the olfactory bulb; **(B)** arrow points to multiple low-signal areas in the olfactory bulb.

Among LCOD patients, 22.9, 45.7, and 28.6% had normal signal intensity, hyperintense signals (Figure 2A), and hypointense signals (Figure 2B), respectively, while among NCPVOD patients, these proportions were 37.1, 11.4, and 48.6%, respectively. There were no significant differences in the proportions of abnormal signals between the two groups. When grouped by OB signal scores (Table 3), between different abnormal OB signal scores group demonstrated no significant differences in the total TDI score, VAS score, or disease duration (*p* > 0.05).

### 3.6 OB volume

**Figure 3:**
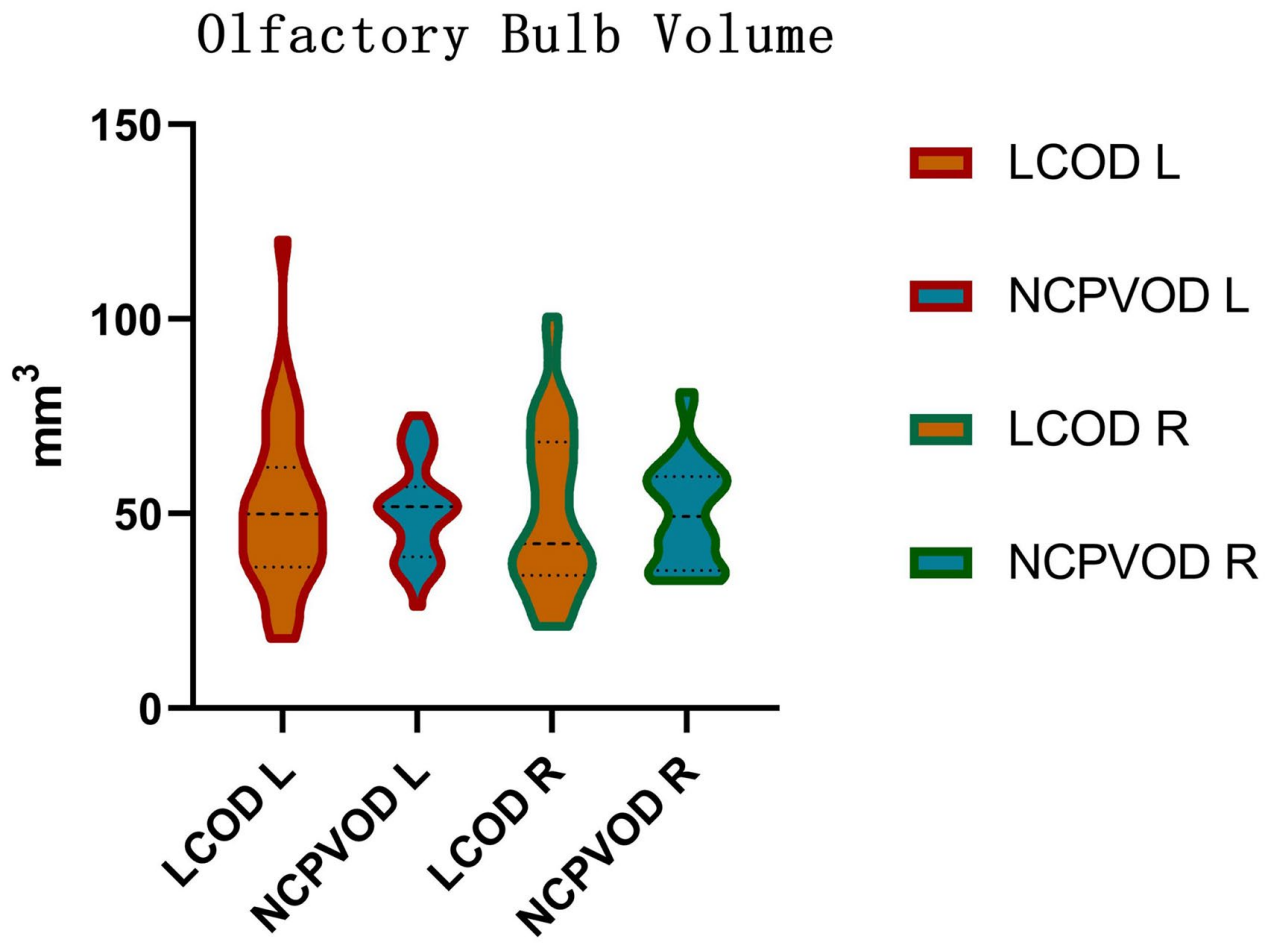
Olfactory bulb volume. Right (R) and left (L) olfactory bulb volumes in the LCOD and NCPVOD groups.

Using 58 mm^3^ (29) as the threshold value for normal bilateral OBs, the proportions of patients with LCOD and NCPVOD with at least one side of the olfactory bulb volume below normal were 24 patients (68.6%) and 27 patients (77.1%), respectively, and the difference was not significant. The OB volume in the LCOD group was 51.28 ± 21.11 mm^3^ (left) and 50.84 ± 21.14 mm^3^ (right); Among the NCPVOD patients, the left and right OB volumes were 50.87 ± 12.48 mm^3^ and 49.84 ± 13.22 mm^3^, respectively (Figure 3). There were no significant differences in the OB volume or the percentage of patients with reduced volumes between the two groups (*p* > 0.05).

### 3.7 OB to rectus gyrus distance, OB to orbital gyrus distance, and relative position of the OBs to the optic nerve

There were no significant differences in the OB to orbital gyrus or rectus gyrus distances between the two groups (*p* > 0.05; Table 4). There was no significant interaction effect of side and group (*p* = 0.985;*p* = 0.683; Table 5).

**Table 4:** Comparison of OB to RG distance, OB to OG distance, and relative position of OB to optic nerve in LCOD and NCPVOD.

	COVID	NCPVOD	df	Effect size	t/Z	*p*
OS depth (mm)						
L	8.48 ± 1.59	7.93 ± 1.54	68	0.175	t = 1.466	0.147
R	9.30 (8.16,10.44)	8.52 (7.28,9.61)	68	0.201	Z = 1.687	0.026
OB to RG distance (mm)						
L	2.83 ± 1.75	3.57 ± 1.73	68	0.220	t = −1.785	0.079
R	2.82 ± 1.51	3.55 ± 1.76	68	0.211	t = −1.866	0.066
OB to OG distance (mm)						
L	5.44 ± 1.80	5.64 ± 1.71	68	0.058	t = 0.482	0.631
R	5.43 ± 1.42	5.39 ± 1.97	68	0.011	t = 0.093	0.926
OB to ONRP (mm)	0 (0.00, 0.98)	0 (0.00, 0.93)	68	0.037	Z = 0.307	0.312

OS, Olfactory sulcus; OB, Olfactory bulb; RG, Rectus gyrus; OG, Orbital gyrus; ONRP, Optic nerve relative position.

**Table 5:** ANOVA results for olfactory sulcus depth, OB to RG distance and OB to OG distance.

	SS	df	Ms	*F*	*p*	η^2^
OS depth						
Side × group	0.364	1	0.364	0.123	0.727	0.001
Group	14.827	1	14.827	4.996	0.027	0.035
Side	11.043	1	11.043	3.721	0.056	0.027
OB to RG distance						
Side × group	0.001	1	0.001	0.000	0.985	0.000
Group	19.063	1	19.063	6.651	0.011	0.047
Side	0.005	1	0.005	0.002	0.967	0.000
OB to OG distance						
Side × group	0.505	1	0.505	0.168	0.683	0.001
Group	0.235	1	0.235	0.078	0.781	0.001
Side	0.662	1	0.662	0.220	0.640	0.002

OS, Olfactory sulcus; OB, Olfactory bulb; RG, Rectus gyrus; OG, Orbital gyrus.

The distance between the OB and optic nerve was 0 mm (0.00, 0.98) in the LCOD group and 0 mm (0.00, 0.93) in the NCPVOD group, and the difference between the groups was not significant (*p* > 0.05; Table 4). In 42 patients (60.0%), the bilateral OBs and optic nerves were on the same horizontal plane.

### 3.8 Olfactory sulcus depth

The olfactory sulcus (OS) depth in LCOD patients was 8.48 ± 1.59 mm (left) and 9.14 ± 2.00 mm (right), whereas in NCPVOD patients, they were 7.93 ± 1.54 mm and 8.39 ± 1.73 mm, respectively. There was no significant interaction effect of side and group (*p* = 0.727). There was a significant main effect between the tow groups (*p* = 0.027; Table 5). There was a significant difference in the right olfactory sulcus depth between the two groups (*p* = 0.026), but not in the left sulcus depth. Overall, the right olfactory sulcus depth was greater than the left olfactory sulcus depth (*p* = 0.003).

### 3.9 Correlations between MRI findings and TDI and VAS scores

**Figure 4:**
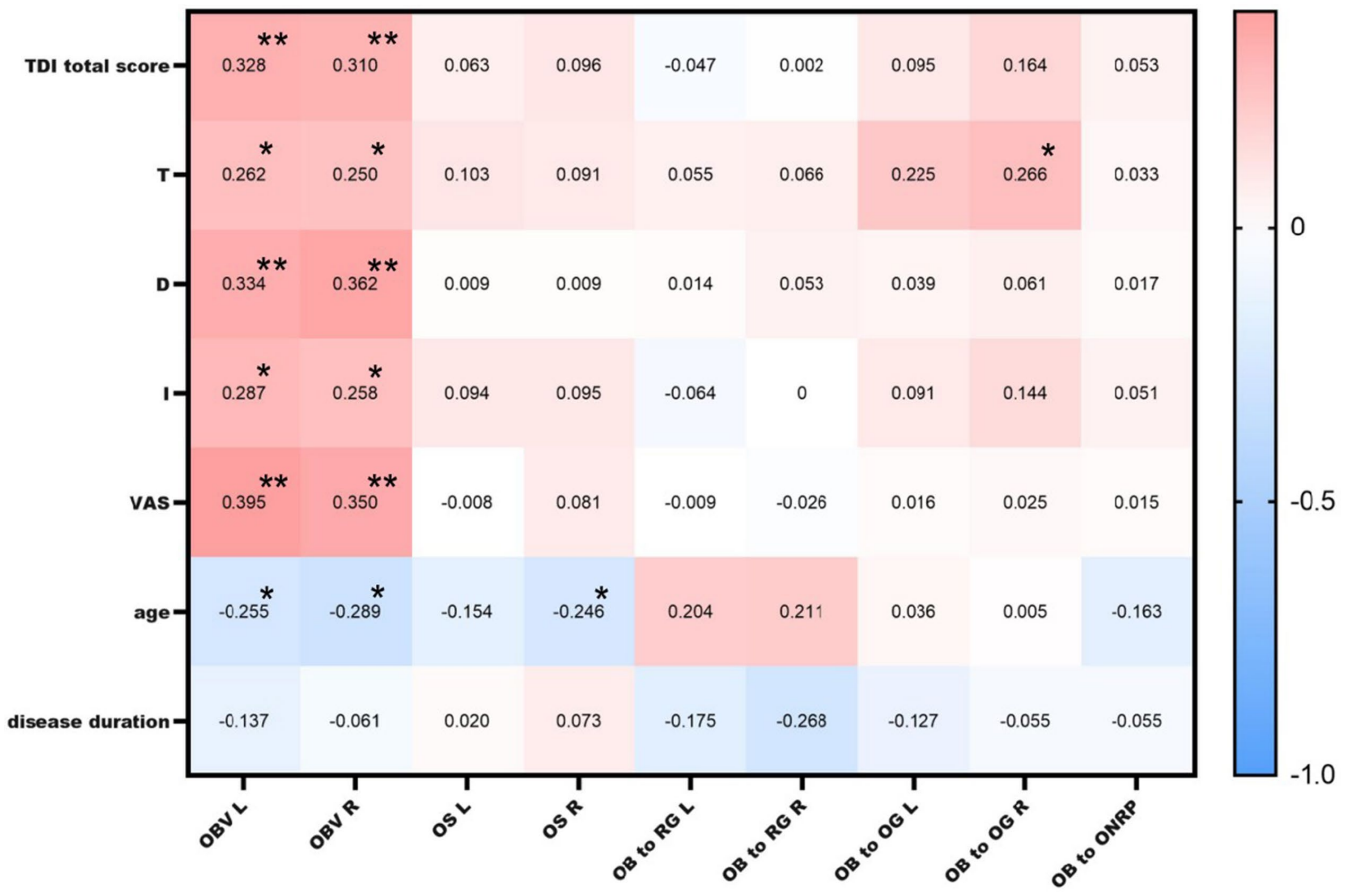
Correlations between MRI findings and TDI and VAS scores. OBV L: left olfactory bulb volume; OBV R: right olfactory bulb volume; OS L: left olfactory sulcus depth; OS R: right olfactory sulcus depth; OB to RG L: left olfactory bulb to rectus gyrus distance; OB to RG R: right olfactory bulb to rectus gyrus distance; OB to OG L: left olfactory bulb to orbital gyrus distance; OB to OG R: right olfactory bulb to orbital gyrus distance; * indicates a significant correlation at the 0.05 level; ** indicates a significant correlation at the 0.01 level.

The total TDI score was positively correlated with the bilateral OB volumes (left: r = 0.33, *p* = 0.006; right: r = 0.31, *p* = 0.009). The olfactory VAS score was also positively correlated with the bilateral OB volumes (left: r = 0.39, *p* = 0.001; right: r = 0.35, *p* = 0.003). Using 58 mm^3^ (29) as the critical threshold for OB volume, there was a significant difference in the total TDI score between the groups (p_left_<0.001; p_right_ = 0.013). OB volume was negatively correlated with age (left: r = −0.25, *p* = 0.033; right: r = −0.29, *p* = 0.015; Figure 4).

A positive correlation was found between the distance from the right OB to the orbital gyrus and the T score (r = 0.266, *p* = 0.026), but no correlation was detected between the OB to optic nerve distance or the OB to orbital gyrus or rectus gyrus distance and the TDI score, age, or disease duration. Moreover, there was no correlation between any of these distances and the TDI or VAS score (*p* > 0.05).

The right olfactory sulcus depth was negatively correlated with age (r = −0.25, *p* = 0.04). There was no significant correlation between the TDI score and left or right olfactory sulcus depth (Figure 4).

## 4 Discussion

This study systematically compared the MR imaging indices of the olfactory pathway, with a focus on the OC and OB, in LCOD and NCPVOD patients. OC opacification was detected on MRI in 80.0% of LCOD patients, and their OC opacification scores were higher than those of NCPVOD patients, which was the main finding of the present study. This suggesting that OC inflammation may be a central mechanism of COVID-19-related olfactory dysfunction.

Higher OC opacity scores in LCOD patients align with prior studies linking OC inflammation to conductive olfactory dysfunction (18, 32). Viral invasion of the OC causes secondary inflammatory changes leading to mucosal oedema and subsequent narrowing of the OC, resulting in odour obstruction and difficulty in reaching the olfactory sulcus (33), leading to conductive olfactory impairment. Moreover, the SARS-CoV-2 virus mobilizes a local immune response and activates inflammatory pathways, functionally affecting olfactory sensory neurons and other olfactory cells through the release of specific cytokines and chemokines in the olfactory mucosa (34) and causing olfactory sensory neuron death (35) and sensory olfactory damage. These two factors may be the main mechanisms by which COVID-19 causes olfactory impairment.

In both LCOD and NCPVOD groups, OB volume reduction was commonly observed. Although no significant differences were found in OB volume between the two groups, this aligns with recent findings indicating minimal OB volume difference in COVID-19 patients compared to controls (36). Previous studies have reported OB atrophy in individuals with olfactory dysfunction (37–39), and autopsy data provided by Chetrit et al. (40) have confirmed the impaired central olfactory site in COVID-19 patients. These findings are consistent with our current observations, but the present study uniquely demonstrated a positive association between OB volume and olfactory function in patients with olfactory dysfunction after viral infection, providing evidence that OB volume may serve as a potential biomarker for olfactory recovery.

In this study, abnormal signals of the OBs were observed in 74.3 and 60% of patients with LCOD and NCPVOD, respectively. OB signal intensity changes, as reported in longitudinal studies of COVID-19 anosmia, suggest transient inflammation that may resolve as smell recovers, indicating a reversible inflammatory process (40). Altunisik et al. (38) reported that significant alterations in olfactory bulb signaling were found in patients with olfactory disorders compared to healthy individuals. However, no significant abnormal signal alterations were found in patients with COVID-19 anosmia after normalization of the olfactory bulb signals to measure processing in another study (37).

We observed a greater right OS depth in LCOD patients, potentially reflecting a lateralized structural alteration associated with COVID-19. In functional olfactory imaging studies, it has been shown that the right olfactory centre has a higher level of activation in right-handed people than the left olfactory centre (41). Further studies by Zang et al. (42) have shown that handedness has no significant effect on olfactory sulcus depth, however, that the right sulcus is significantly greater than the left sulcus and that the olfactory system has right-sided laterality. The depth of the right olfactory sulcus has been shown to be correlated with olfactory function scores in one study (12). As the right olfactory system is more sensitive to changes than the left olfactory system, the difference in the depth of the right olfactory sulcus was significant between LCOD and NCPVOD patients, perhaps suggesting that COVID-19 has a unique effect on the structural integrity of the olfactory pathway.

A novel aspect of this study was the introduction of the spatial relationships between the OB, orbital gyrus, rectus gyrus, and optic nerve. Although these new parameters did not significantly differ between the two OD groups, the correlation between the OB to orbital gyrus distance and the olfactory threshold score (r = 0.266, *p* = 0.026) suggests a potential direction for future research. These spatial measurements can help elucidate the anatomical basis of olfactory function and may serve as potential predictive tools.

This study has several limitations. First, a healthy control group was not included, limiting direct comparisons with unaffected individuals. Although we referenced established MRI data for healthy populations, future research with a control group could validate and expand upon our findings. Additionally, the study’s relatively small sample size may reduce the generalizability of our results, especially given the variability in COVID-19 disease severity and duration among patients. Another limitation is the reliance on cross-sectional MRI assessments; longitudinal imaging could offer insights into the progression and potential reversibility of observed olfactory structural changes over time. Due to the manual segmentation method, the results may introduce operator bias. We measured as little bias as possible by using two operators, but in the future there is hope that this manual error will be reduced by automated identification techniques. OB volume was not normalized to total intracranial volume (TIV), which may introduce variability based on individual brain sizes. Future studies consider incorporating OB volumes normalized to TIV to enhance the robustness of the findings.

Our findings underscore the role of OC inflammation in LCOD. Existing studies, including Pendolino et al. (43) and Saussez et al. (44, 45) report mixed efficacy of corticosteroids for COVID-19-related olfactory dysfunction. Although anti-inflammatory treatments play a role in the recovery of olfactory impairment, further studies are needed to investigate the best treatment options and safety. Future research should investigate the efficacy of targeted treatments that focus on reducing OC inflammation, as this may help restore olfactory function more effectively. Additionally, OB atrophy as a common outcome in viral-induced olfactory dysfunction. This study’s results are consistent with recent imaging studies, affirming the need for targeted diagnostic approaches for prolonged COVID-19 anosmia, MRI-based assessments of OB and OC abnormalities may serve as valuable diagnostic and prognostic tools for identifying patients likely to benefit from early intervention and monitoring recovery progression.

## Data Availability

The original contributions presented in the study are included in the article/supplementary material, further inquiries can be directed to the corresponding author.
